# Induced Phases of New H-bonded Supramolecular Liquid Crystal Complexes; Mesomorphic and Geometrical Estimation

**DOI:** 10.3390/molecules25071549

**Published:** 2020-03-28

**Authors:** Rua B. Alnoman, Hoda A. Ahmed, Mohamed Hagar, Khulood A. Abu Al-Ola, Bedor Sh. Alrefay, Bashayer A. Haddad, Raghad F. Albalawi, Razan H. Aljuhani, Lama D. Aloqebi, Shoaa F. Alsenani

**Affiliations:** 1 Chemistry Department, College of Sciences, Taibah University, Yanbu 30799, Saudi Arabia; rua-b-n@live.co.uk (R.B.A.); tu3753281@taibahu.edu.sa (B.S.A.); Hadad5040@gmail.com (B.A.H.); Rero-_2011@hotmail.com (R.F.A.); Aa-rr14@hotmail.com (R.H.A.); lama_aloqebi@hotmail.com (L.D.A.); sho3a3ah@gmail.com (S.F.A.); 2 Faculty of Science, Department of Chemistry, Cairo University, Cairo 12613, Egypt; 3 Faculty of Science, Chemistry Department, Alexandria University, Alexandria 21321, Egypt; 4 Chemistry Department, College of Sciences, Al-Madina Al-Munawarah, Taibah University, Al-Madina 30002, Saudi Arabia; Kabualola@taibahu.edu.sa

**Keywords:** supramolecular H-bonding complexes, 4-Alkoxyphenyliminobenzoic acid, induced phase, DFT calculations, mesomorphic Schiff base liquid crystals

## Abstract

New five rings architecture of 1:1 supramolecular hydrogen bonded (H-bonded) complexes were formed between 4-(2-(pyridin-4-yl)diazenyl-3-methylphenyl 4-alkoxybenzoates and 4-n-alkoxyphenyliminobenzoic acids. Mesomorphic and optical behaviors of three systems designed complexes were investigated by differential scanning calorimetry (DSC) and polarizing optical microscopy (POM). H-bonded interactions were confirmed via FT-IR spectroscopy. Computational calculations were carried out by density functional theory (DFT) estimation for all formed complexes. Experimental evaluations were correlated with the theoretical predictions and results revealed that, all prepared complexes possessing enantiotropic tri-mesophases with induced smectic C (SmC) and nematic temperature ranges. Moreover, DFT predicted for all formed supramolecular complexes possessing a non-linear bent geometry. Moreover, the π–π stacking of the aromatic rings plays an important role in the mesomorphic properties and thermal stabilities of observed phases. The energy changes between frontier molecular orbitals (HOMO and LUMO) and the molecular electrostatic potential (MEP) of the designed complexes were discussed and related to the experimental results.

## 1. Introduction

Supramolecular approaches have a strong impact on various daily life device applications such as display devices, sensors, etc. These types of materials have an essential role in material science developments. The concept of Supramolecular liquid crystals has been successively applied for potential wide applications in scientific and technological fields [1,2,3,4,5]. Nowadays, thermotropic hydrogen-bonded (H-bonded) liquid crystal complexes show great potential and are attracting interest [6,7,8,9,10,11,12,13,14,15]. Many hydrogen-bonded systems based on pyridine (as hydrogen acceptor) and carboxylic acid (as the hydrogen donor) have been studied and characterized extensively [16,17,18,19,20,21,22]. Supramolecular H-bonded liquid crystals (SMHBLCs) combine of supramolecular chemistry with mesogenic behavior principles. The induced mesophase or mesophase stabilities in such systems are most often attributed to the enhancement of the molecular anisotropy of the resulting complexes with respect to that of the individual components [23,24,25,26,27,28]. The mesomorphic behavior of a wide variety of SMHBLC dimers have been reported by varying the spacer length and the molecular structures of the individual components [6]. There is a different approach in which can control the molecular shape using H-bonding [23,24,25,26,27,28]. Most of the SMHLCs studied are based on the rod-like intermolecular H-bonding [10,12,13,14,29,30,31] and there is a lot of interest towards the formation of angular supramolecular hydrogen-bonded liquid crystals [9]. On the other hand, the Schiff base and azo moiety are of special interest due to their ability for trans/cis isomerization upon UV light irradiations [7,32,33,34,35]. In their supramolecular Schiff base/azo -based LCs, via H-bonding interactions, the rigid core is lengthened, and thus induces the mesomorphic behavior that may not occur in the individual components [11,36]. Further, the induction of liquid crystalline criteria is also accompanied by strong hydrogen bonds formed between the interacting components. This would accordingly be enhanced by the change in polarity and/or polarizability of both interacting components.

**Scheme 1:**
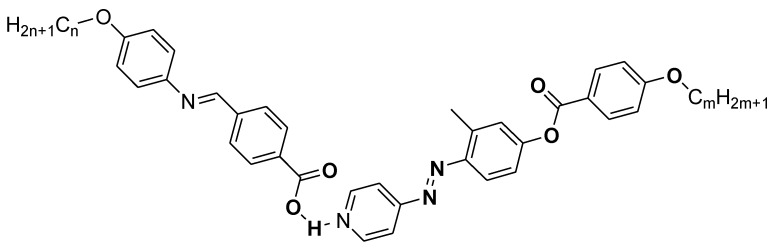
1:1 Supramolecular H-bonded complexes, **I An/Im**; n = 6,8 and 16; m = 8,10,12 and 16.

The goal of the present study is to investigate experimentally and theoretically the thermal behavior resulting from intermolecular H-bonding interactions between the 4-alkoxyphenylimino benzoic acids (**An,** Scheme 1) and the lateral methyl, 4-(2-(pyridin-4-yl)diazenyl-3-methylphenyl 4-alkoxybenzoates, (**Im)** [37]. Therefore, we designed all possible 1:1 supramolecular complex **An/Im** with various flexible terminal alkoxy chains in order to study the effect of proportionating lengths of flexibles wings on the mesophase behavior. Today, density functional theory (DFT) became an excellent performance tool correlated with experimental evidences. Moreover, the estimated structural geometry and thermal data, such as the dipole moment, polarizability, and energies gab between the frontier molecular orbitals of the prepared compounds are also studied. Further comparison is made between the present system architectures and previously reported complexes to investigate the effect of changing the mesogenic moiety on estimated experimental parameters.

## 2. Results and Discussion

### 2.1. Fourier Transform Infrared Spectroscopy (FT-IR):

**Figure 1:**
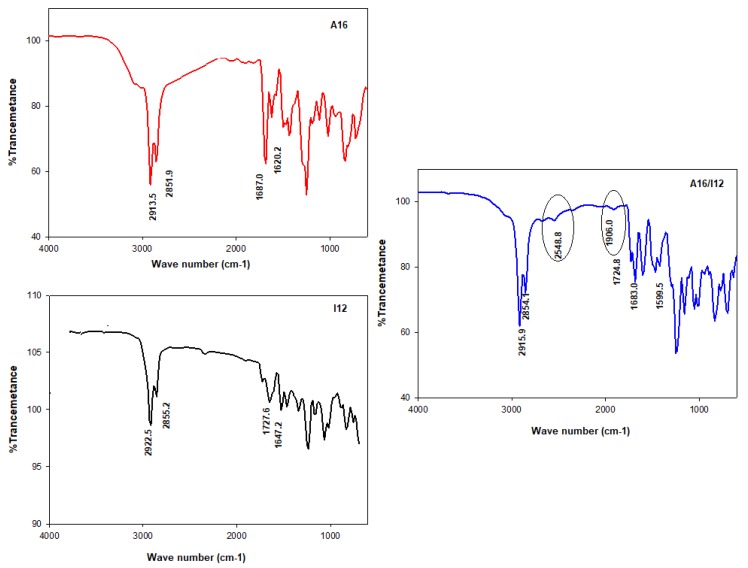
FT-IR spectrum of the acid **A16**, **I12** and their Supramolecular H-bonded Complexes (SMHBCs) **A16/I12**.

FT-IR spectra were recorded to prove the construction of the SMHBCs. The spectral data were measured for complexes **A16/I12** as well as their individual components, Figure 1.

It is well known that [8,10,28,38,39,40,41] a major verification of the SMHBC formation is the existence of three Fermi vibrational resonating bands of OH group, **A**-, **B**- and **C**-types [11,28]. The **A**-type Fermi band lay under the C–H stretching vibrational bands at 2915 to 2854 cm^−1^. However, **B**-type at 2548 cm^−1^ can be assigned to the fundamental in-plane bending vibrational band of the O–H. Finally, the C-type Fermi vibrational band owing to the interaction between the fundamental stretching vibration of the OH and its overtone due to the torsional impact and appeared at 1906 cm^−1^ [40].

### 2.2. Mesomorphic Studies of 1:1 Molar Supramolecular Complexes

**Figure 2:**
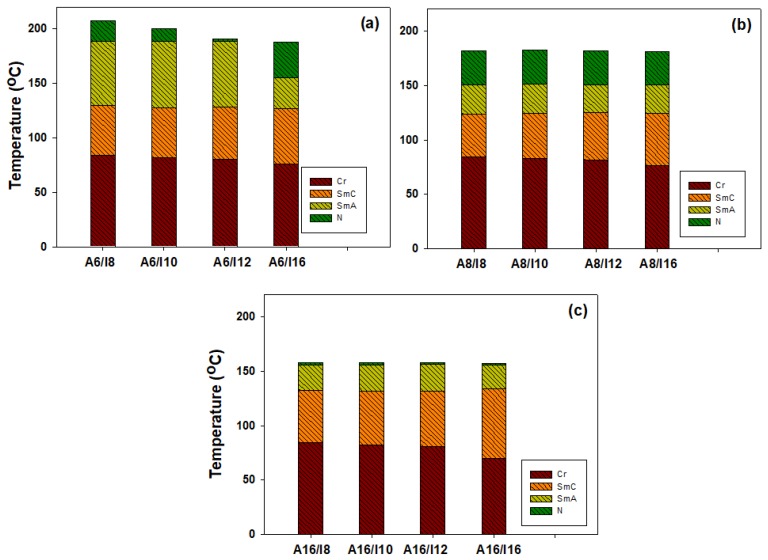
Effect of the alkoxy-chain length (**m**) of the azopyridine-based derivative (**Im**) on mesophase behavior of the 1:1 SMHBCs (**a**) **A6**/**Im**; (**b**) **A8**/**Im**; (**c**) **A8**/**Im**.

**Figure 3:**
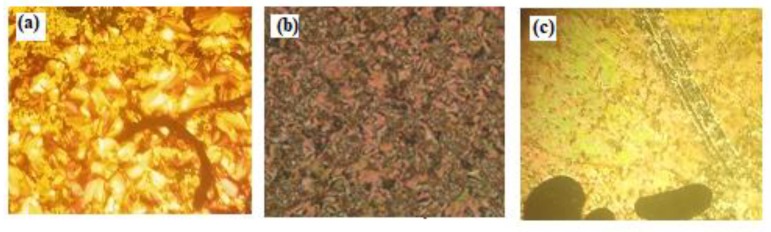
Polarizing optical microscopy (POM) textures upon heating of SMHBC **A16**/**I10** (**a**) SmC phase at 120.0 °C; (**b**) SmA phase at 143.0 °C; and (**c**) nematic phase at 157.0 °C.

**Figure 4:**
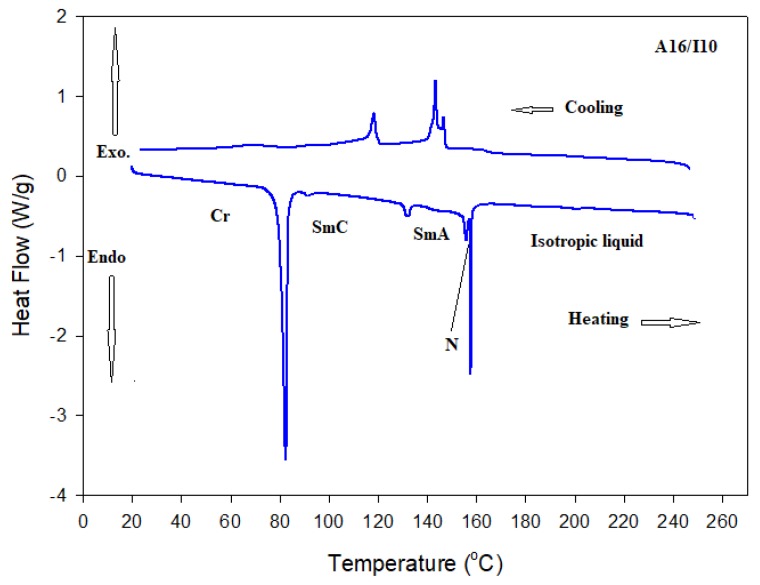
DSC thermograms upon second heating/cooling cycles of SMHBC **A16**/**I10**.

Herein, the mesomorphic behavior and optical transitions of the prepared lateral-CH_3_ supramolecular H-bonded complexes (**An/Im**) were analyzed and investigated by DSC and POM. POM was used to detect phase textures of the liquid crystal phases exhibited by the complexes and all observations were verified by the DSC measurements. Resulted data of the transition temperatures and their associated enthalpy as well as normalized entropy of mesophase transitions for the all characterized SMHBCs **An/Im**, are collected in Table 1 which derived from DSC measurements. Moreover, the transition temperatures of the investigated binary complexes were graphically represented in Figure 2. Figure 2 shows the effect of the terminal flexible-chain length of the lateral methyl base component on the mesophase behavior. All prepared SMLHB complexes showed enantiotropic mesomorphism during heating and cooling cycles. Enantiotropic property meaning that high stable mesophase which observed in both heating and cooling scans of thermograms. The POM investigations for textures were confirmed triple mesophases, smectic C phase then smectic A mesophase followed by nematic phase (Figure 3**)**. Thermograms of DSC upon the second heating/cooling scans of supramolecular complexes **A16/I10** are shown in Figure 4, as an example.

It should be noticed that the phase behavior of the prepared 4-alkoxy phenyliminobenzoic acids **An** exhibits smectic A phase (SmA) with narrow nematic phase range in case of short terminal flexible chain length (n = 6 and 8), while showing only the SmA phase for long chains (n = 16). [42] The lateral-CH_3_azopyridines **Im** exhibits only the smectic C phase (SmC) with lower stability. [37] Therefore, it was interesting to study the mesophase behavior of the complexes resulting from mixing Schiff base acid derivatives **An** and the monotropic azopyridines **Im**.

Table 1 and Figure 2 data revealed that enantiotropic triple-phases are observed for all investigated 1:1 mixtures. In addition, the thermal stabilities of smectogenic phase slightly linear in relation to increment the wings flexible chain length. Moreover, the melting transition temperatures have irregular trends. Despite the fact that the pure 4-n-alkoxyphenylimino benzoic acids (**An**) exhibit a smectic A phase transition with relatively high temperatures and a very small range of nematic phase (N) depending on their chain lengths [42], while the azopyridines (**Im**) possess poor SmC mesophase [37], the present H-bonded complexes display a pronounced thermal stability when heated due to the H-bonding between their components leads to an elongation of the rigid segment of the individual compounds. Therefore, the mesophases of all prepared 1:1 SMHBCs (**An/Im**) observed showed induced SmC and nematic phases with broad temperature ranges. For the first series of complexes **A6/Im**, while the Schiff base acid component **A6** is dimorphic exhibiting SmA and narrow range of nematic mesophase, the resulted binary complexes possess slightly broad mesophase temperature ranges which covering all chain lengths of the alkoxy-base components. The SmC stability varies regularly with all lengths of alkoxy-base terminals. SmA and N phase stabilities show a decrement with the increment of flexible chain lengthens. The higher value of SmC range observed for **A6/I16** (~50.8 °C) and the lower range value for **A6/I10** (~46.0 °C). Conversely, for SmA mesophase range the higher value for **A6/I10** (~61.0 °C) and the lower value for **A6/I16** (~2.8 °C). The nematic phase range for **A6/ I16** has a maximal value of ~32.8 °C and minimal one at **A6/I12** ~1.8 °C. Moreover, the mesomorphic range decrease in order **A6/ I8** > **A6/I10** > **A6/I16** > **A6/I12.** In the case of the second series of complexes **A8/Im** also the Schiff base acid component **A8** is dimorphic exhibiting SmA and narrow nematic range. The same observations of the first series were obtained in resulted complexes **A8/Im** which possesses slightly wide mesomorphic ranges with induced SmC and N phase ranges covering all chain lengths of the alkoxy-base components **Im**. The SmC, SmA and nematic phases stabilities vary regularly with all lengths of alkoxy-base terminals (Figure 2). The maximal value of SmC range observed for **A8/I16** was ~47.7 °C and the minimal range value for **A8/I8** was ~39.5 °C. For the SmA mesophase range the maximal value observed for **A8/I10** was ~27.2 °C and the minimal value for **A8/I12** was ~26.1 °C. The induced nematic mesophase range for **A8/I8** had a maximal value ~31.4°C and the minimal value of **A8/I16** ~30.1 °C. Furthermore, the mesomorphic range increase with the increase of the alkoxy chain **m** in the order **A8/I16** > **A8/I12** > **A8/I10** > **A8/I8.** For the third series of supramolecular complexes **A16/Im,** the 4-n-hexadecyloxyphenylimino benzoic acid **A16** is monomorphic and possesses only the SmA phase. Then formed complexes **A16/Im** exhibiting induced N phase covering all alkoxy chain lengths. The SmC, SmA and nematic phases stabilities vary linearly with all lengths of alkoxy- base wings (Figure 2). The maximal SmC range value observed for **A16/I16** was ~63.6 °C) and the minimal range value for **A16/I8** was ~48.1 °C. In the case of SmA mesophase range the maximal value observed for **A16/I12** was~24.3 °C and the minimal value for **A16/I16** was ~21.8 °C. The induced nematic mesophase range observed for all 1:1 mixtures and has a maximal value of ~2.1 °C for **A16/I8 = A16/I10 and** the minimal value of ~1.4 °C obtained for **A16/I12**. Thus, the length and core of the present molecules were found to be more dominant on the stability of the observed N phase. It would be concluded that the increment of the molecular anisotropy in the SMHBCs promotes broadening of nematic phases that agree with the previous work [43] which revealed that the increase of the mesogenic part length enhancement the stability of nematic phases. Finally, the mesomorphic range is enhanced with the increment of the alkoxy chain lengths **(m**) in order **A16/I16** > **A16/I12** > **A16/I10** > **A16/I8**.

**Scheme 2:**
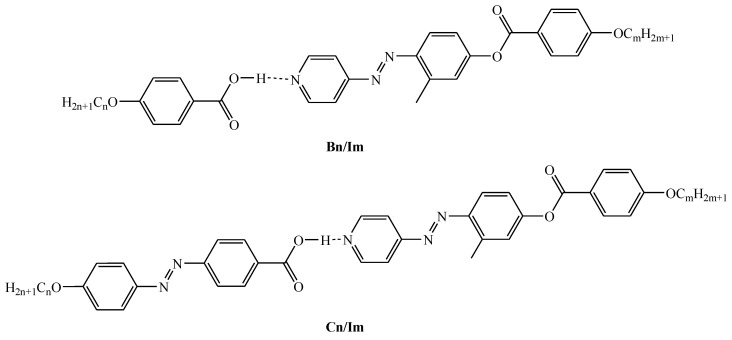
Supramolecular H-bonded complexes **Bn/Im** and **Cn/Im**.

In order to study the effect of changing the core moiety of acid molecules on the mesophase behavior of 1:1 molar mixtures SMHBCs, comparison was constructed between the mesophase stabilities of the present Schiff base acid complexes (**An/Im**) and the previous 4-n-alkoxybenzoic acids SMHBCs (**Bn/ Im**) [37] as well as our reported work 4-n-alkoxyphenylazobenzoic acids complexes (**Cn/ Im**) [23] as a function of terminal alkoxy-chain length (see Scheme 2). The study revealed that increasing the length and rigidity of the acid mesogenic core resulted in an increase of the stabilities and mesomorphic ranges of both the smectic and nematic phases. In addition, Induced nematic phase observed for present complexes-based Schiff base moiety.

**Figure 5:**
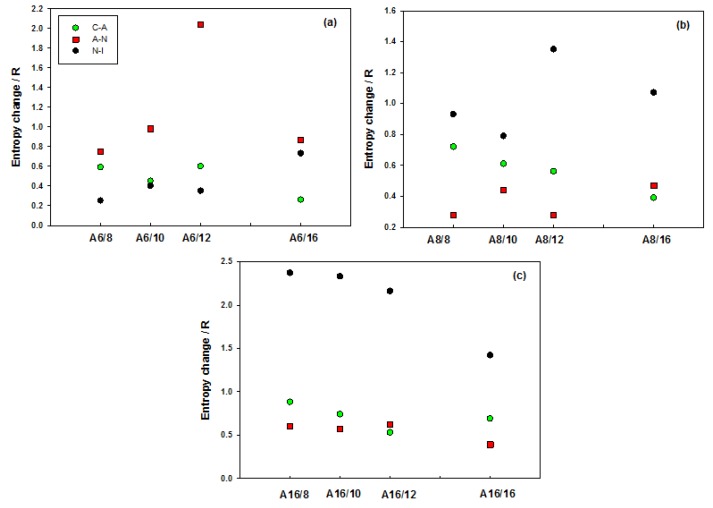
Normalized entropy changes as a function of alkoxy chain length **m** for SMHBCs **An/Im**; (**a**) n = 6; (**b**) n = 8 and (**c**) n = 16; C-A = smectic C to smectic A transition; A-N = smectic A to nematic phase transition; N-I = nematic to isotropic liquid transition.

Normalized entropies of transitions of the smectic C–to-smectic A and smectic A-to–nematic and nematic –to- isotropic liquid were estimated from DSC results for all prepared the SMHBCs (An/Im). The results are tabulated in Table 1 and graphically represented in Figure 5. As seen from Table 1 and Figure 5, independent of the length of the flexible terminal chains (n or m), all entropies of transitions (*ΔS*) are of irregular trends. That results in agreement with previous findings [36]. Moreover, the irregular dependency indicates that the length of the terminal substituents, whether on the acid molecule (n) or the base component (m), led to random Δ*S* values due to the irregular change of lateral adhesion upon the increase of the total molecular length [44].

### 2.3. DFT Theoretical Calculations

#### 2.3.1. Molecular Geometry of SMHBCs

**Figure 6:**
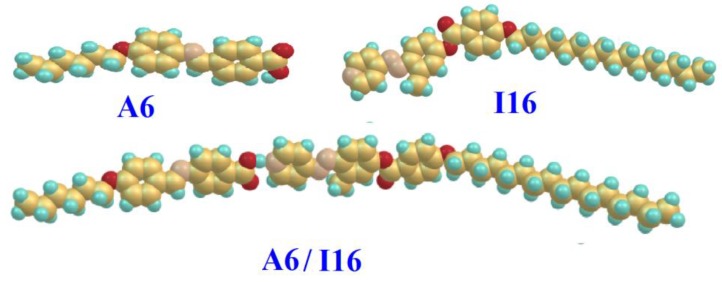
The calculated molecular geometry of the base **I16**, the acid **A6** and their SMHBCs, **A6/I16.**

The structural geometry of the prepared SMHBCs (**A6/I16**) has been predicted by DFT theoretical calculations at the basis set B3LYP 6-311G (Supplementary Materials). The absence of imaginary frequencies is an evidence of the structural stability of all H-bonded complexes. Figure 6 showed the optimal structural geometry of the base (**I16**) and Schiff base acid **A6** as well as their H-bonded complexes **A6/I16**.

Although both individual components are in a planar geometry, their SMHCs are non-linear and none co-planar. As shown from Figure 6, the supramolecular complexes **An/Im** exhibiting a non-linear bent geometry.

In order to investigate the impact of the chain length either of the base **Im** and/or the acid **An** on the mesomorphic behavior of the SMHBCs, the dimensional parameters (D width, L length) and the aspect ratios (L/D) were calculated by estimating the diameter of the collision of these SMHB complexes parameters. Although the chain length of the alkoxy terminal of the carboxylic acid and the base increases the dimensional parameters with considerable value, the aspect ratio has no significant with the change of the chain terminals. As shown from Table 2, the mesophase rage, as well as the nematic stability, decreases with increases of either the alkoxy chain of the acid or the base even with the constant of the aspect ratio. This result could be illustrated in terms of the dilution of the aromatic mesogenic core with increases of the chain length. The higher the percentage of the aromatic core of the liquid crystalline compounds with longer chain length the more strength of the lateral interaction, where, the higher aromatic ratio permits the maximum degree of packing of the molecules. Moreover, the change of the chain length of the SMHBCs affects the competitive intermolecular lateral and terminal interactions. Since these compounds showed three mesophases, the mesophase stability of each enhanced mesophase and their range are highly impacted by the chain length of the acid as well as the base. The shorter chain length of the base and/or the acid resulted in the increment of the lateral interaction to enhance the more ordered smectic phases (SmC and SmA) range and stability. On the other hand, the longer the terminal chains make a dilution of the aromatic mesogenic core to enhance terminal aggregation over lateral stacking, consequently, the less ordered nematic mesophase range will be increased.

#### 2.3.2. Thermal Parameters

**Figure 7:**
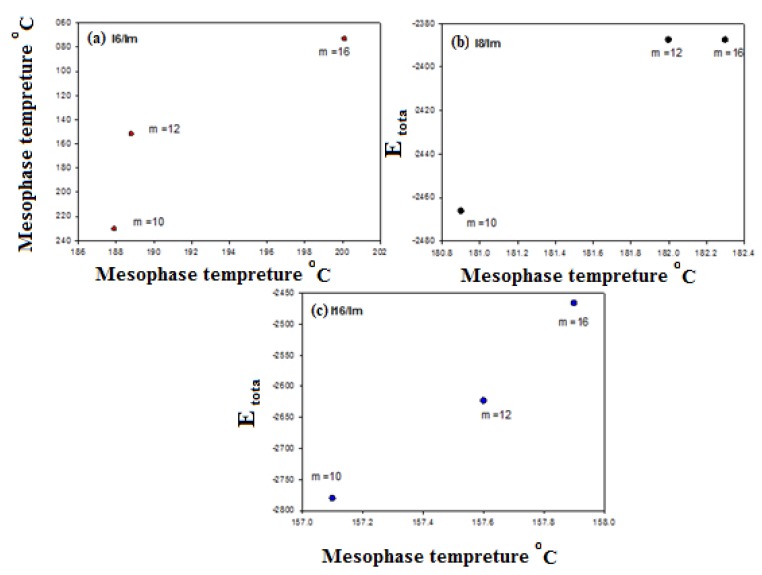
Relationship between the sum of the electronic and thermal energies of H-bonded complexes **An/Im**; (**a**) n = 6; (**b**) n = 8 and (**c**) n = 16.

The predicted thermodynamic parameters were estimated with the same method at the same set for all prepared H-bonded complexes (**An/Im)** and the data were summarized in Table 3. Obviously, the thermal energy of the SMHBCs decreases with increasing of the chain length of the acid and/or the base. This can be illustrated in terms of the higher degree of packing of the molecules at longer chain lengths and resulting in higher stability of the liquid crystalline molecules. At longer chain lengths more Van der Waal interaction between the alkoxy chains and consequently, lower the thermal energy of SMHCs. As shown in Figure 7, the longer the alkoxy chains the higher the stability of the SMHBCs with lower the mesophase range and stability. The decrement of the stability range could be explained in terms of the impact of the terminal chains, as the length increases the degree of terminal aggregation increases and resulting in the range decrement.

#### 2.3.3. Frontier Molecular Orbitals and Polarizability

**Figure 8:**
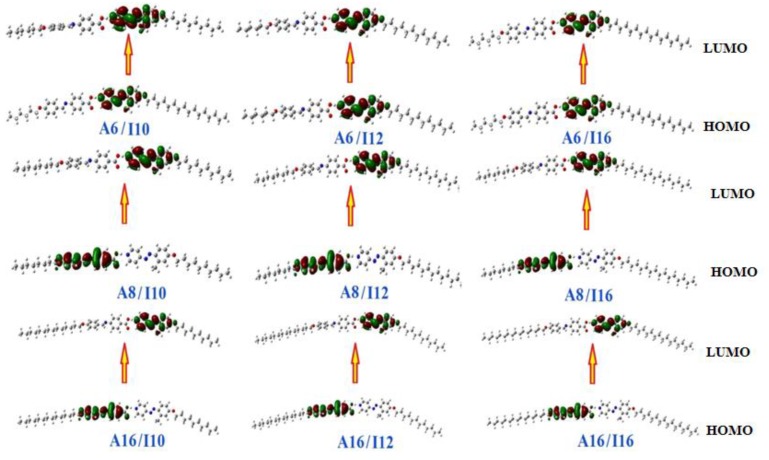
The estimated plots for frontier molecular orbitals of angular-shaped conformer of SMHB complexes, **An/Im**.

Figure 8 showed the estimated plots for frontier molecular orbitals (FMOs), highest occupied molecular orbitals (HOMO) and lowest unoccupied molecular orbitals (LUMO) of the prepared SMHDCs **An/Im**. It is obvious that the electron densities FMOs of HOMO are mainly localized on the base **Im**. However, the electron densities are shifted to the Schiff acid for the formation of the LUMOs. As shown from Table 4, the length of the terminal chains has no impact neither in the level of the FMOs nor the energy gap between them. It is well known that the gap between FMOs can be used as tool to expect of the capacity of the electron to transfer between the FMOs during the processes of electronic excitation. Moreover, it can be used in the calculation the parameters that expect the polarizability of the molecules such as the global softness (**S**) = **1 /ΔE** and chemical hardness **η**, these parameters show the sensitivity of the liquid crystals for the photoelectric effects. The better global softness of the compounds leads to better polarizability as well as the photoelectric sensitive. As shown from Table 4, the polarizability of H-bonding liquid crystal increases with the chain length either for the base or the acid moieties.

#### 2.3.4. Molecular Electrostatic Potential (MEP)

**Figure 9:**
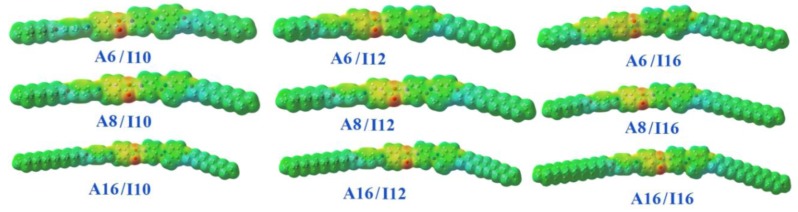
Molecular electrostatic potentials (MEP) for SMHBCs, **An/Im**.

The charge distribution map for SMHBCs **An/Im** was calculated with the same method at the same basis sets according to molecular electrostatic potential (MEP) (Figure 9). The negatively charged atomic sites (the red region) were expected to be localized on H-bonded part of carboxylate moiety of the Schiff acid, while the moiety of the base as well as the alkoxy chains showed the least negatively charged atomic sites (blue regions). As shown from Figure 9**,** the alkoxy chain length of the SMHBCs **An/Im** does not affect neither the orientation nor the amount of the charge distribution map, this could illustrate the similar mesophases observed for all lengths

## 3. Experiment

### Preparation of Supramolecular H-bonded Complexes (SMHBCs), An/Im

**Scheme 3:**
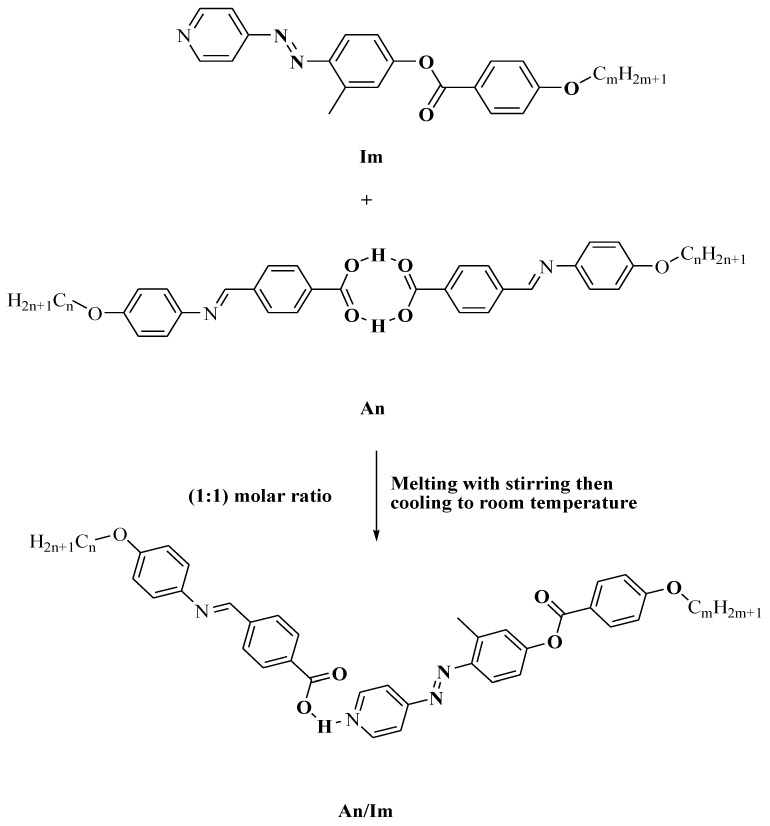
Preparation of 1:1 supramolecular H-bonded complexes (**An/Im**).

SMHBCs (**An/Im**), are prepared by mixing 1:1 molar ratio corresponding component by melting with stirring till an intimate blend. The formed complexes were cold to room temperature (Scheme 3).

## 4. Conclusions

Herein, we have reported new three-homologue series of 1:1 supramolecular H-bonded complexes have five aromatic rings. Mesomorphic and optical characterizations were carried out by DSC and POM. The intermolecular H-bond formation of complexes was confirmed by observation of Fermi-bands via FT-IR spectroscopic technique. DFT estimation was performed to calculate the thermal and structural parameters for prepared SMHBCs. Results revealed that:

1. All prepared SMHBCs exhibit high thermal stability enantiotropic tri-mesophases are, SmA, SmC and N phases.
2. Induced SmC and nematic temperature ranges were observed.
3. Geometrical parameters estimations of the prepared complexes are highly affected by the electronic nature of the molecular shape as well as the flexible chain lengths.
4. Inclusion of a phenylimino moiety in the acid component increases the stabilities of both the smectic C and nematic mesophases.
5. The entropy changes are varying irregularly with either of the terminal chain length **n** or **m**.
6. The DFT estimations showed that SMHCs are non-linear and none co-planar.
7. The mesophase rage, as well as the mesophase stability, decreases with increases of either the alkoxy chain of the acid or the base even with the constant of the aspect ratio.
8. The decrement of the stability range has been explained in terms of the impact of the terminal chain interactions.
9. The polarizability of H-bonding liquid crystal increases with the chain length either for the base or the acid moieties.

## Figures and Tables

**Table 1:** Phase transition temperatures (°C), enthalpy of transitions (kJ/mol) and normalized transition entropy for the supramolecular complexes **An**/**Im.**

Compounds	*T*_Cr-C_	*∆H*_Cr-C_	*∆S*_Cr-C_	*T*_C-A_	*∆H*_C-A_	*∆S*_C-A_	*T*_A-N_	*∆H*_A-N_	*∆S*_A-N_	*T*_N-I_	*∆H*_N-I_	*∆S*_N-I_
A6/I8	83.8	63.30	21.33	129.9	1.99	0.59	188.8	2.89	0.75	207.6	1.00	0.25
A6/I10	81.7	63.99	21.69	127.7	1.49	0.45	188.7	3.75	0.98	200.1	1.57	0.40
A6/I12	80.6	56.98	19.37	128.5	1.99	0.60	187.0	7.82	2.04	188.8	1.32	0.35
A6/I16	76.3	67.80	23.34	127.1	0.86	0.26	155.1	3.10	0.87	187.9	2.81	0.73
A8/I8	84.2	42.34	14.25	123.7	2.36	0.72	150.5	0.99	0.28	181.9	3.53	0.93
A8/I10	82.8	55.24	18.67	124.3	2.01	0.61	151.5	1.56	0.44	182.3	2.99	0.79
A8/I12	81.2	65.32	22.17	124.8	1.85	0.56	150.9	0.99	0.28	182.0	5.09	1.35
A8/I16	76.4	78.81	27.12	124.1	1.29	0.39	150.8	1.64	0.47	180.9	4.04	1.07
A16/I8	84.3	43.14	14.52	132.4	2.96	0.88	155.5	2.13	0.60	157.6	8.47	2.37
A16/I10	82.5	49.95	16.89	132.0	2.48	0.74	155.8	2.02	0.57	157.9	8.35	2.33
A16/I12	80.6	48.02	16.33	131.9	1.79	0.53	156.2	2.22	0.62	157.6	7.73	2.16
A16/I16	69.9	78.70	27.59	133.5	2.32	0.69	155.3	1.39	0.39	157.1	5.07	1.42

Abbreviations: Cr-C = crystal to smectic C phase transition; C-A = smectic C to smectic A transition; A-N = smectic A to nematic phase transition; N-I = nematic to isotropic liquid transition.

**Table 2:** Dimensional parameters, aspect ratios and mesophases range of SMHBCs **An/Im.**

Parameter		A6/I10	A6/I12	A6/I16	A8/I10	A8/I12	A8/I16	A16/I10	A16/I12	A16/I16
Dimensions Å	Width (D)	9.3	9.7	10.5	9.4	10.1	10.8	10.8	12.3	11.2
	Length (L)	49.4	51.9	56.9	51.9	56.5	59.4	59.1	60.9	69.5
Aspect ratio (L/D)		5.3	5.3	5.3	5.4	5.4	5.5	5.6	5.5	5.5
*T*_Cr-C_		81.7	80.6	76.3	82.8	81.2	76.4	82.5	80.6	69.9
*∆S*_Cr-C_		21.69	19.37	23.34	18.67	22.17	27.12	16.89	16.33	27.59
*T*_C-A_		127.7	128.5	127.1	124.3	124.8	124.1	132	131.9	133.5
*∆S*_C-A_		0.45	0.6	0.26	0.61	0.56	0.39	0.74	0.53	0.69
*T*_A-N_		188.7	188.8	155.1	151.5	150.9	150.8	155.8	156.2	155.3
*∆S*_A-N_		0.98	2.04	0.87	0.44	0.28	0.47	0.57	0.62	0.39
*T*_N-I_		200.1	187	187.9	182.3	182	180.9	157.9	157.6	157.1
*∆S*_N-I_		0.4	0.35	0.73	0.79	1.35	1.07	2.33	2.16	1.42
*∆T*_SmC_		46.0	47.9	50.8	41.5	43.6	47.7	49.5	51.3	63.6
*∆T*_SmA_		61.0	60.3	28.0	27.2	26.1	26.7	23.8	24.3	21.8
*∆T*_N_		11.4	11.8	32.8	30.8	31.1	30.1	2.1	1.4	1.8
*∆T* Mesophase range Total		118.4	106.4	111.6	99.5	100.8	104.5	75.4	77.0	87.2
*T*_C_ Mesophase stability		200.1	187.0	187.9	182.3	182.0	180.9	157.9	157.6	157.1

**Table 3:** Thermal parameters (Hartree/Particle) of both conformers of the H-bonded complexes **An/Im**.

Parameters	A6/I10	A6/I12	A6/I16	A8/I10	A8/I12	A8/I16	A16/I10	A16/I12	A16/I16
E_corr_	0.833	0.890	0.947	1.062	1.090	1.118	1.118	1.233	1.347
ZPVE	−2073.353	−2151.920	−2230.487	−2387.621	−2426.905	−2466.189	−2466.188	−2623.320	−2780.457
E*_tot_*	−2073.304	−2151.868	−2230.432	−2387.561	−2387.561	−2466.125	−2466.125	−2623.250	−2780.383
H	−2073.303	−2151.867	−2230.431	−2387.560	−2387.560	−2466.125	−2466.124	−2623.250	−2780.382
G	−2073.451	−2152.021	−2230.592	−2387.734	−2387.734	−2466.307	−2466.306	−2623.450	−2780.592

Abbreviations; ZPVE: Sum of electronic and zero-point energies; E_tot_: Sum of electronic and thermal energies; H: Sum of electronic and thermal enthalpies; G: Sum of electronic and thermal free energies.

**Table 4:** Frontier molecular orbitals (FMO) Energies a.u., polarizability, α, and dipole moment μ (Debye) of geometrical conformers of HBSMCs, **An/Im**.

Parameters	A6/I10	A6/I12	A6/I16	A8/I10	A8/I12	A8/I16	A16/I10	A16/I12	A16/I16
E_LUMO_	−0.1063	−0.1063	−0.1063	−0.1063	−0.1063	−0.1061	−0.1061	−0.1062	−0.1063
E_HOMO_	−0.2138	−0.2138	−0.2138	−0.2138	−0.2138	−0.2138	−0.2140	−0.2136	−0.2137
ΔE_HOMO-LUMO_	0.1075	0.1075	0.1075	0.1075	0.1075	0.1077	0.1079	0.1074	0.1074
μ Total	6.2859	6.3105	6.3341	6.2601	6.2411	6.3085	6.2227	6.2474	6.2673
Polarizability α	626.03	649.80	697.15	650.22	685.95	721.67	721.24	769.10	816.95

## Supplementary Materials

The following are available online. Scheme S1: Synthesis of 4-[4(alkoxy)phenylimino)methyl]benzoic acid (An), Figure S1: 1H NMR of 4-[4-(hexyloxy)phenylimino)methyl]benzoic acid (A6), Figure S2: C13 NMR of 4-[4-(hexyloxy)phenylimino)methyl]benzoic acid (A6).
