# Supplementary material for: Induced Phases of New H-bonded Supramolecular Liquid Crystal Complexes; Mesomorphic and Geometrical Estimation
Source: Molecules. 2020 Mar 28;25(7):1549. doi: 10.3390/molecules25071549 (PMC7180916; doi:10.3390/molecules25071549)
Supplement: Supplementary file 1 [file molecules-25-01549-s001.pdf]

molecules

Supplementary Data

Induced Phases of New H-bonded Supramolecular Liquid Crystal Complexes; Mesomorphic and Geometrical Estimation

Rua B. Alnoman [1], Hoda A. Ahmed [2,*], Mohamed Hagar [1,3,*], Khulood A. Abu Al-Ola [4], Bedor Sh. Alrefay [1], Bashayer A. Haddad [1], Raghad F. Albalawi [1], Razan H. Aljuhani [1], Lama D. Aloqebi [1] and Shoaa F. Alsenani [1]

[1] Chemistry Department, College of Sciences, Taibah University, Yanbu 30799, Saudi Arabia; rua-b-n@live.co.uk(R.B.A.); tu3753281@taibahu.edu.sa(B.Sh.A.); Hadad5040@gmail.com(B.A.H.); Rero-_2011@hotmail.com(R.F.A.); Aa-rr14@hotmail.com(R.H.A.); lama_aloqebi@hotmail.com(L.D.A.); sho3a3ah@gmail.com(S.F.A.)

[2] Faculty of Science, Department of Chemistry, Cairo University, Cairo 12613, Egypt

[3] Faculty of Science, Chemistry Department, Alexandria University, Alexandria, 21321, Egypt

[4] Chemistry Department, College of Sciences, Al-Madina Al-Munawarah, Taibah University, Al-Madina 30002, Saudi Arabia; Kabualola@taibahu.edu.sa

* Correspondence: ahoda@sci.cu.edu.eg(H.A.A.); mhagar@taibahu.edu.sa(M.H.)

1. Experimental

1.1. Materials

4-Hexyloxyaniline, 4-octyloxyaniline, 4-hexadecyloxyaniline were purchased from Sigma Aldrich (Germany). The 4-formylbenzoic acid and ethanol were purchased from Aldrich (Wisconsin, USA).

1.2. Synthesis

Compounds **An** were prepared according to the following scheme:

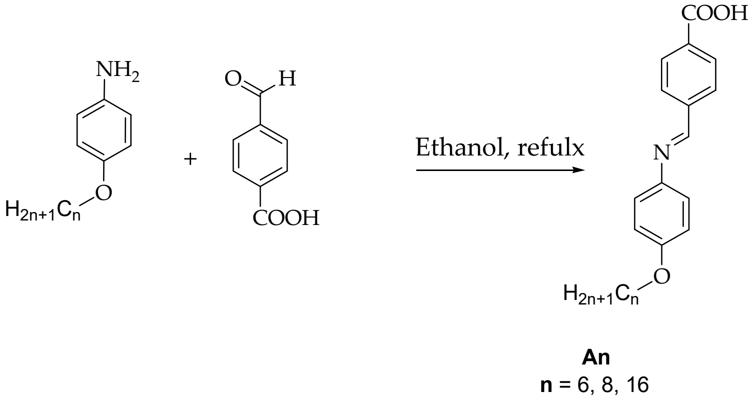

Scheme 1. Synthesis of 4-[4(alkoxy)phenylimino)methyl]benzoic acid (**An**).

1.2.1. Synthesis of 4-[4(alkoxy)phenylimino)methyl]benzoic acid (**An**)

An equimolar amount of 4-formylbenzoic acid (610 mg, 4.1 mmol) and 4-hexyloxyaniline (790 mg, 4.1 mmol) in ethanol (10 mL) were refluxed for two hours. The reaction mixture was allowed to cool and the separated product was filtered. The obtained solid was recrystallized from ethanol [1–3].

Yield: 93.0%; mp 190.0 °C, FTIR (ύ, cm^{-1}): 2930–2864 (CH_2 stretching), 1678 (C=O), 1616 (C=N),1597 (C=C), 1490 ($C–O_{Asym}$), 1235 ($C–O_{Sym}$). ^1H NMR (400 MHz, $CDCl_3$) δ 10.03 (s, 1H, CH=N), 8.13 (d, *J* = 8.5 Hz, 2H, ArH), 7.97 (d, *J* = 8.5 Hz, 2H, ArH), 7.40 (d, *J* = 8.4 Hz, 2H, ArH), 6.99 (d, *J* = 8.6 Hz, 2H, ArH), 4.05 (t, *J* = 6.5 Hz, 2H, $CH_3(CH_2)_3CH_2CH_2$), 1.91 – 1.76 (m, 2H,$CH_3(CH_2)_3CH_2CH_2$), 1.58–1.56 (m, 6H, $CH_3(CH_2)_3CH_2CH_2$), 0.88 (t, *J* = 6.6 Hz, 3H, $CH_3(CH_2)_3CH_2CH_2$). ^{13}C NMR (101 MHz, $CDCl_3$) δ 191.62, 169.63, 159.57, 139.62, 134.29, 130.78, 129.60, 124.37, 115.44, 115.42, 68.39, 31.55, 29.22, 25.64, 22.60, 14.05. Elemental analyses: Found (Calc.): C, 73.79 (73.82); H, 7.11 (7.12); N, 4.29 (4.30).

1.2.2. Instruments

TA Instruments Co. Q20 Differential Scanning Calorimeter (DSC; USA) were using for calorimetric measurements. The DSC was calibrated using the melting temperature and enthalpy of indium and lead. DSC investigation was carried out for small samples (2–3 mg) placed in aluminum pans. All measurements were achieved at a heating rate of 10 °C/min in inert atmosphere of nitrogen gas (30 mL/min) and all transition recorded from the second heating scan.

Transition temperatures for the individual components and their 2:1 associated complexes were determined by DSC, and the types of the mesophase identified by a standard polarized optical microscope (POM, Wild, Germany) attached with Mettler FP82HT hot stage. The temperature is measured by a thermocouple attached to the temperature controller. Measurements were made twice and the results have accuracy in transition temperature within ± 0.2 °C.

1.2.3. Characterizations

Molecular structures of **Im** were checked with thin-layer chromatography using TLC and elemental analyses. The structure was confirmed by FTIR (Nicolet iS 10 Thermo scientific), and ^1H-NMR spectroscopy (Varian EM 350L 300 MHz spectrometer, Oxford, UK).

1.2.4. Computational Method and Calculations

The theoretical calculations for the investigated compounds were carried out by Gaussian 09 software [4]. DFT methods using the B3LYP 6-311G basis set was selected for the calculations. The geometries were optimized by minimizing the energies with respect to all geometrical parameters without imposing any molecular symmetry constraints. The structures of the optimized geometries had been drawn with Gauss View [5]. Moreover, the calculated frequencies were carried out using the same level of theory. The frequency calculations showed that all structures were stationary points in the geometry optimization method with none imaginary frequency.

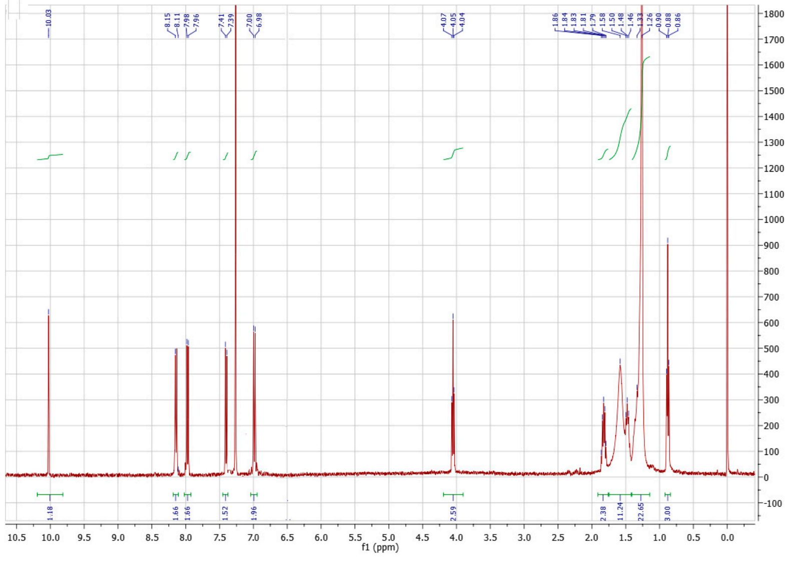

Figure S1. ^1H NMR of 4-[4-(hexyloxy)phenylimino)methyl]benzoic acid (A6).

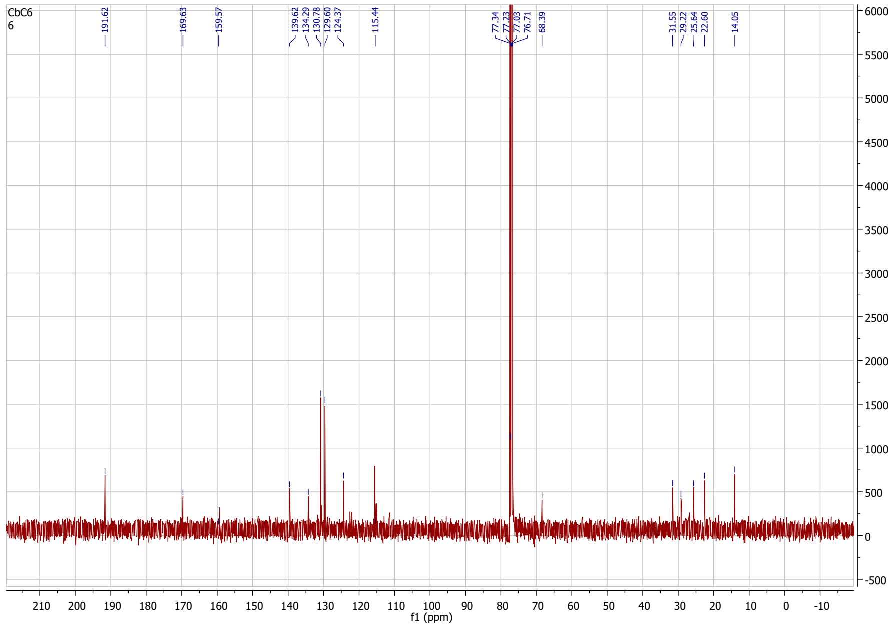

Figure S2. C^{13} NMR of 4-[4-(hexyloxy)phenylimino)methyl]benzoic acid (A6).

References.

1. Weissflog, W.; Lischka, C.; Diele, S.; Pelzl, G.; Wirth, I.; Grande, S.; Kresse, H.; Schmalfuss, H.; Hartung, H.; Stettler, A. Banana-shaped or rod-like mesogens? Molecular structure, crystal structure and mesophase behaviour of 4, 6-dichloro-1, 3-phenylene bis [4-(4-n-subst.-phenyliminomethyl) benzoates]. *Mol. Cryst. Liq. Cryst. Sci. Technol. Sect. A. Mol. Cryst. Liq. Cryst.* **1999**, *333*, 203–235.
2. Niori, T.; Yamamoto, J.; Yokoyama, H. New mesogenic compounds exhibiting a thermotropic optically isotropic phase. *Mol. Cryst. Liq. Cryst. Sci. Technol. Sect. A. Mol. Cryst. Liq. Cryst.* **2001**, *364*, 843–850.
3. Nafee, S.S.; Hagar, M.; Ahmed, H.A.; El-Shishtawy, R.M.; Raffah, B.M. The synthesis of new thermal stable schiff base/ester liquid crystals: A computational, mesomorphic, and optical study. *Molecules* **2019**, *24*, 3032.
4. Frisch, M.; Trucks, G.; Schlegel, H.B.; Scuseria, G.; Robb, M.; Cheeseman, J.; Scalmani, G.; Barone, V.; Mennucci, B.; Petersson, G. Gaussian 09, revision a. 02, *Gaussian Inc.:* Connecticut, CT, USA, **2009,** 200.
5. Dennington, R.; Keith, T.; Millam, J. GaussView, version 5; *Semichem Inc.:* Shawnee Mission, KS, USA, 2009.
